# Brensocatib (an oral, reversible inhibitor of dipeptidyl peptidase-1) attenuates disease progression in two animal models of rheumatoid arthritis

**DOI:** 10.3389/fimmu.2023.1231047

**Published:** 2023-08-11

**Authors:** Patrick P. McDonald, Franziska Graf Leifer, Jessica Basso, Dan Lasala, Dedong Li, Kuan-Ju Chen, Jimin Zhang, Walter R. Perkins, David C. Cipolla

**Affiliations:** Department of Research, Insmed Incorporated, Bridgewater, NJ, United States

**Keywords:** DPP-1, cathepsin C, neutrophil serine proteases, inflammation, neutrophils, migration

## Abstract

Rheumatoid arthritis (RA) is a painful and incurable disease characterized by chronic joint inflammation and a progressive destruction of cartilage and bone. Although current treatments have improved clinical outcomes for some patients, the high relapse rates and sizeable proportion of non-responders emphasize the need for further research. Arthritic joints are massively infiltrated by neutrophils, which influence inflammatory and immune processes by releasing cytokines, chemokines, eicosanoids, and neutrophil serine proteases (NSPs) – all of which are known to contribute to RA initiation and progression. Active NSPs are generated from zymogens at the promyelocytic stage of neutrophil differentiation under the action of dipeptidyl peptidase 1 (DPP-1) and DPP-1 knockout mice are resistant to the development of arthritis. Thus, DPP-1 inhibition represents a promising therapeutic approach in RA. In this study, we assessed the efficacy of a potent and highly selective DPP-1 inhibitor, brensocatib, in two well established RA models – rat collagen-induced arthritis (CIA) and mouse collagen antibody-induced arthritis (CAIA). In both models, brensocatib at 3 and 30 mg/kg/day significantly reduced bone marrow NSP levels, in keeping with prior pharmacodynamic studies in rodents. More importantly, brensocatib treatment significantly improved disease score at both dosages in both rodent models. In the mouse CAIA model, brensocatib even proved at least as potent as anti-TNF antibodies in diminishing both the histopathological score and neutrophil infiltration into arthritic joints. Together, these results show that brensocatib alters RA disease progression in rodents and supports the need for its further evaluation as a potential therapeutic option, or to complement existing RA treatments.

## Introduction

Rheumatoid Arthritis (RA) is an auto-immune disorder that is characterized by chronic inflammation of the joints. It is a widespread disease (affecting about 1% of the adult population worldwide according to the WHO) that is painful, incapacitating, and currently incurable. RA patients generally experience flares of high intensity symptoms, alternating with periods of remission. To temporarily control acute episodes, non-steroidal anti-inflammatory drugs and/or corticosteroids are commonly employed. However, rheumatologists often prefer to limit the duration of such therapy because of the numerous side effects, which include diminished bone strength and increased susceptibility to infections. On the other hand, disease modifying anti-rheumatic drugs (DMARDs) such as methotrexate are widely used to treat RA patients. These drugs can take weeks or even a few months to become effective and regulate the immune system. By comparison, biologic DMARDs are faster-acting than traditional DMARDs and mostly target the inflammatory process; a prominent example is anti-TNFα antibodies. Although costlier and sometimes more challenging to use due to the immunosuppression that they cause, biologics can dramatically improve the prognosis and function of RA patients, especially those who do not respond to treatment with traditional DMARDs. Nonetheless, all medications currently used to treat RA have significant side effects; there remains a sizeable proportion of RA patients that are non-responders to any available treatment; and even among responders, improvement scores vary between 20-75% (www.arthritis.org). Clearly, better treatments for RA are urgently needed – even for patients that already respond to a given therapy.

Towards this goal, revisiting the cellular and molecular bases underlying RA pathogenesis can offer new perspectives. RA is characterized by ongoing inflammation and cartilage destruction, which are largely mediated by infiltrating leukocytes and their products. In fact, one of the hallmarks of arthritic joints is the massive presence of neutrophils within both the synovial fluid and synovial membrane, particularly at the pannus-erosion boundary (1). Accordingly, several neutrophil products are detected in RA synovial fluid, including IL-1β, IL-17A, CXCL1, CXCL8, TNFα, and LTB4 (1–6). Neutrophil-derived cytokines and chemokines have even proven essential for the initiation and acute phases of arthritis in murine models (7–10). Similarly, citrullinated histone H4 and carbamylated LL-37, which are present on neutrophil extracellular traps (NETs), were recently shown to be targets of autoantibodies found in RA (11, 12) and it is increasingly clear that NETs represent a connection between innate and adaptive immunity in this pathology (1, 11–14). Finally, neutrophils at the pannus-cartilage junction in arthritic joints contribute to matrix degradation through their release of matrix metalloproteinases (e.g. MMP-8, MMP-9) (15) and neutrophil-specific serine proteases (NSPs) such as neutrophil elastase (NE), cathepsin G (CatG), and proteinase 3 (PR3) (16, 17). Incidentally, NSPs are also present on NETs (18, 19); among other things, NET-associated NSPs can process and activate cytokines of the IL-1 family *in vivo* (20) and may thus contribute to the pro-inflammatory actions of NETs in RA. Thus, neutrophils and their products (e.g. cytokines, chemokines, eicosanoids, NETs, NSPs), as well as the cellular machinery controlling their formation, are important elements of RA pathogenesis and as such, represent promising targets for therapeutic intervention. Despite this, there are no approved neutrophil-targeting therapeutic strategies to treat RA.

Among the aforementioned neutrophil products, most of the cytokines, chemokines or eicosanoids are made by several cell types; targeting them would therefore affect far more than neutrophils, as is the case with anti-TNFα antibodies. Conversely, NSPs are mostly restricted to neutrophils and can cause tissue damage either as released free enzymes or as NET components. Whereas inhibitors exist for at least some NSPs, the proteases may act independently or in a partially redundant manner; as a result, it would be preferable to attenuate all NSPs simultaneously with one treatment, prior to their release from the neutrophil granules. In this regard, all three NSPs are synthesized as inactive pro-proteins during granulocyte development (21) and later cleaved into active NSPs during neutrophil maturation in the bone marrow by a single enzyme, dipeptidyl peptidase 1 (DPP-1, also known as cathepsin C) (22, 23). This alone underscores the potential of DPP-1 as a therapeutic target in RA. More compelling evidence stems from the finding that DPP-1 knockout mice are resistant to the development of collagen antibody-induced arthritis (CAIA) (24).

Thus, we were interested in evaluating how brensocatib – an oral, selective, competitive and reversible DPP-1 inhibitor, might attenuate disease progression in animal models of RA. Brensocatib is currently under clinical evaluation in a 52-week Phase III trial in another chronic inflammatory indication, non-cystic fibrosis bronchiectasis (NCFBE; NCT04594369). Data from the WILLOW Phase IIb trial in NCFBE (NCT03218917) demonstrated that brensocatib inhibited NE in humans and led to a better therapeutic outcome (25, 26). We now report that brensocatib downregulates the activity of the main pathophysiologically relevant DPP-1 substrates (e.g. NE, CatG, PR3) in two animal models of RA, and describe the outcomes of brensocatib treatment in these disease models.

## Materials and methods

### Rat collagen-induced arthritis model

Female Lewis rats were immunized on Days 1 and 7 with a collagen emulsion or (for the non-disease group) with vehicle (i.e., 0.9% saline). Female animals were chosen as disease incidence is significantly higher than in males; disease onset is also more rapid in females (27). All collagen-treated animals developed the disease. Brensocatib was given by oral administration (i.e., dissolved in 0.5% hydroxypropyl methylcellulose, 0.5% Tween 80, citrate buffer, pH 3) twice daily, 8 h apart, at 0.15, 1.5, or 15 mg/kg (i.e. 0.3, 3.0, or 30 mg/kg/day). The vehicle control followed the same administration scheme as for the brensocatib arms in an identical volume of 10 ml/kg. Dexamethasone was given by oral administration once daily at 0.3 mg/kg. For both brensocatib and dexamethasone, dosing was initiated on day 1 and continued through the end of study on day 30 or day 31, to stagger sample collection burden equally between groups.

Disease score measurements (clinical score and paw thickness) were performed on days 1, 7, 11, 14, 16, 18, 21, 23, 25, 28, and 30. Scoring was considered (0) Normal; (1) Erythema and mild swelling confined to the mid-foot (tarsals) or ankle/wrist joint, or digits; (2) Erythema and mild swelling extending from the ankle/wrist to the mid-foot/paw (2 segments); (3) Erythema and moderate swelling extending from the ankle/wrist to the metatarsal joints (2 segments); (4) Erythema and severe swelling encompassing the ankle/wrist, foot/paw, and digits. Body weight was measured every 4 days.

### Mouse CAIA model

Male DBA/1 mice were immunized on day 0 with intravenous (i.v.) injection of 5-clone mouse type II collagen antibody (Chondrex, Inc; 1.5 mg/dose), except in the Non-Disease/Vehicle group, which were left untreated. Male animals were chosen as females are significantly less susceptible, possibly because estrogen decreases disease incidence (28). On day 3, all mice that received the collagen antibody cocktail also received an intraperitoneal injection of 25 μg LPS; all animals thusly treated developed the disease. Brensocatib was administered orally at 3 and 30 mg/kg/day twice daily (BID) from day -10 to day 1 and once daily (QD) from day 2 to day 20. The vehicle control followed the same administration scheme as for the brensocatib arms in an identical volume of 5 ml/kg. Dosing began on day -10 and continued through the end of the study on day 21. Anti-TNF-α was administered 3x/week at 5 mg/kg. Dosing of anti-TNFα began on day 0 and continued through day 21.

Mice were evaluated for arthritis every other day from day 0 to day 21. The evaluation involved scoring each paw separately on a scale of 0 to 4, where 0 was normal and 4 equated to severe swelling resulting in rigidity of the joint such that the animal would not put weight on that limb. Each animal therefore had a cumulative score of up to 16 for each day of evaluation. Additionally, paw swelling was evaluated on days -1, 7, 14, and 20 by measurement of paw thickness in all 4 paws using a digital caliper. Body weight was measured daily.

### NSP quantitation

NE, PR3, and CatG kinetic activity assays were conducted as previously described (29), with slight modifications. Briefly, bone marrow lysates were assayed in enzymatic kinetic assays using the following substrates (final concentrations are indicated): for NE, 100 µM N-methoxysuccinyl-Ala-Ala-Pro-Val-7-amido-4-methylcoumarin (Sigma, St. Louis, MO; excitation/emission at 350/450 nm); for PR3, 40 µM (7-methoxycoumarin-4-yl)acetyl-lysyl-(picolinoyl)-Tyr-Asp-Ala-Lys-Gly-Asp-N-3-(2-4-dinitrophenyl)-2-3-diaminopropyonyl-NH2) (GenScript, Piscataway, NJ; excitation/emission at 340/430 nm); and for CatG, 200 µM N-succinyl-Ala-Ala-Pro-Phe p nitroanilide (Sigma; absorbance at 405 nm). Fluorescence or absorbance was quantified using a Synergy microplate reader (BioTek; Winooski, VT). The specific NSP activity in each sample was calculated as total activity minus the activity measured in the presence of a specific NSP inhibitor – elastase inhibitor (Abcam) for NE, sivelestat (Abcam) for PR3, and cathepsin G inhibitor I (Cayman Chemical; Ann Arbor, MI) for CatG. Active NSP concentrations were interpolated based on their activities relative to the standard curves created using active human NE protein (Sigma), active human PR3 protein (Sigma), and active human CatG protein (Sigma), respectively. Due to the unavailability of commercial mouse NE, PR3, and CatG proteins, the corresponding human proteins were used since their catalytic properties are predominantly conserved across species. A portion of each cell lysate sample was also set aside for protein quantitation using a Pierce BCA Protein Assay Kit (Thermo Fisher). NSP activities were normalized for the cell lysate protein concentrations.

### Data analysis method for kinetic NSP assays

Data analysis was performed as previously described (29). Briefly, the linear portion of the kinetic slopes was either (1) visually determined and calculated using Excel’s slope formula; or (2) automatically determined and calculated using an internally developed Excel macro. Standard curves were created using the standard slope values and their respective known concentrations. The unknown sample concentrations were then calculated using the second-degree polynomial line of best fit formula from the appropriate standard curves.

### Histopathology analyses

Hind paw samples (ankle with attached paw) fixed in 10% neutral buffered formalin were decalcified, then paraffin embedded in the frontal plane in individual blocks. Tissue slices were stained for H&E; cartilage damage was assessed by monitoring loss of toluidine blue staining (proteoglycan), along with chondrocyte loss and/or collagen disruption; bone resorption was assessed by monitoring the percentage area of subchondral bone being affected. Tissue slices were similarly processed for routine immunohistochemical (IHC) staining using myeloperoxidase (MPO) or Ly6G antibodies.

### Statistical analyses

All statistical analyses were performed as indicated in the figure legends, using Prism 9 software (GraphPad Software, San Diego, CA, USA).

## Results

### Effect of brensocatib on NSP activities in animal RA models

**Figure 1:**
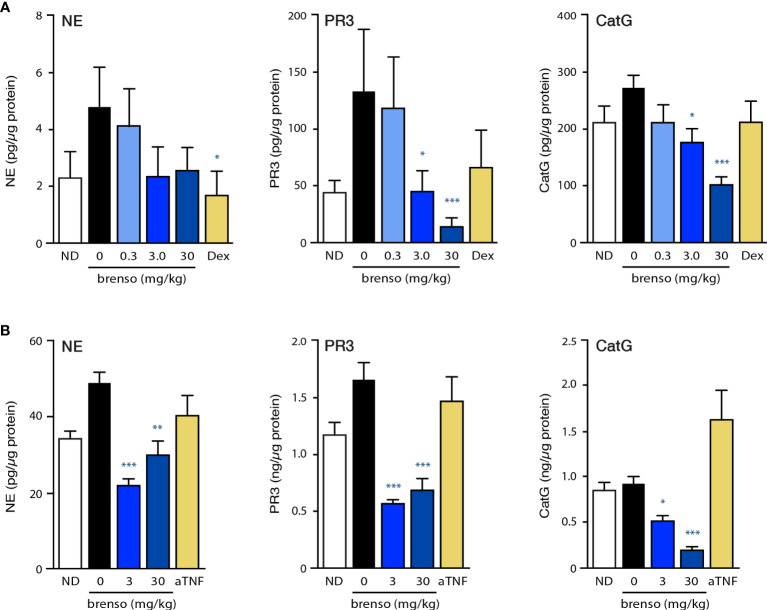
Effect of brensocatib on classical DPP-1 substrates in two animal models. **(A)** Rats were treated in the CIA model and samples were analyzed as described in *Methods*. Briefly, rats were immunized twice with a collagen emulsion to induce arthritis or with vehicle (non-disease group). In animals developing arthritis, brensocatib was given orally at 0.3, 3.0 and 30 mg/kg/day; as a control, a group was given dexamethasone (“Dex”) orally once daily at 0.3 mg/kg. Bone marrow was isolated on day 30 or 31 and NSP activities were assessed (NE, neutrophil elastase; PR3, proteinase 3; CatG, cathepsin G). Mean ± SEM of at least 10 animals per group. **(B)** Mice were treated in the CAIA model and samples were analyzed as described in *Methods*. Briefly, mice were immunized on day 0 with i.v. injection of a collagen antibody cocktail or with vehicle (non-disease group). On day 3, all mice that received the collagen antibodies also received an injection of 25 μg LPS i.p.; non-disease control animals received an injection of vehicle instead. Vehicle control or brensocatib at 3 or 30 mg/kg/day was given orally from day -10 to day 1 BID, and from day 2 to day 20 QD. Anti-TNFα (“aTNF”) was administered 3x/week at 5 mg/kg, starting on day 0 and continued through day 21. Bone marrow was isolated on day day 21 and NSP activities were assessed. Mean ± SEM of at least 8 animals per group, except for non-diseased mice (5 animals). Statistical differences in this figure were determined using Kruskal-Wallis test with Dunn’s multiple comparisons *post-hoc* test. *p< 0.05; **p< 0.01; ***p< 0.001, relative to diseased animals receiving vehicle only.

We first investigated to what extent DPP-1 inhibition by brensocatib would affect the pharmacodynamic activity of classical DPP-1 substrates (NE, CatG, PR3) in the two RA animal models. For this purpose, we measured NSP activities in the bone marrow. As shown in **Figure 1A**, NSP activities for the negative control in the rat CIA model were elevated following disease onset compared to levels observed in non-diseased animals. Brensocatib treatment restored NSP activities to baseline (i.e., those of non-diseased animals) levels or below, and this was most evident at the two highest inhibitor dosages (**Figure 1A**). In the mouse CAIA model, disease onset again resulted in an increase in bone marrow NE and PR3 levels for the animals in the negative control, and this was reversed in animals receiving both brensocatib dosages (**Figure 1B**). Even though bone marrow CatG levels were not augmented following disease induction, brensocatib treatment caused CatG activity to dip significantly below baseline levels, showing that the inhibitor worked as expected.

### Effect of brensocatib on clinical parameters in two animal models of RA

**Figure 2:**
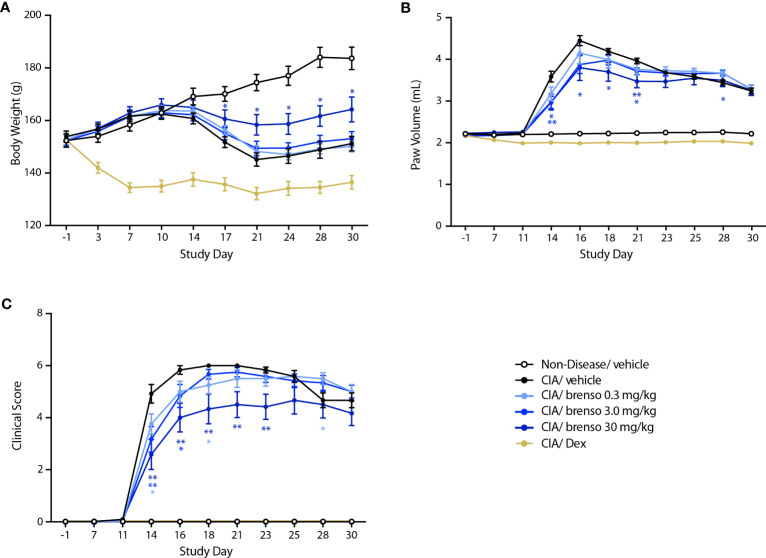
Effect of brensocatib on body weight and disease parameters in the rat CIA model. Rats were treated as described in **Figure 1** for arthritis induction and inhibitor administration. The following parameters were monitored: **(A)** body weight; **(B)** paw thickness; **(C)** clinical score (sum of the two hind paws). Mean ± SEM of at least 10 animals per group. Statistical differences were determined using multiple t test comparisons and a false discovery rate of 1% as determined by the two-stage set-up method of Benjamini, Krieger, and Yekutieli. *p< 0.05; **p< 0.01 relative to diseased animals receiving vehicle only.

We next investigated how brensocatib might affect disease progression in the well-established rat CIA model. RA was induced by two collagen immunizations one week apart with twice daily brensocatib treatments at 0.3, 3.0, and 30 mg/kg/day which were initiated concurrently with the first collagen immunization and lasted for 30 or 31 days. Disease levels were evaluated throughout the study. As shown in **Figure 2A**, treatment with the highest brensocatib dose (30 mg/kg/day) resulted in a significant recovery of weight loss compared to CIA/vehicle alone, starting at day 17. By comparison, the average body weight of the dexamethasone group was much lower than that of diseased animals, as commonly observed in this model. Additionally, a significant reduction in inflamed paw volume was observed in the 3.0 and 30 mg/kg/day brensocatib groups between days 14 and 21, relative to diseased animals (**Figure 2B**). Finally, the clinical score showed significant reductions compared to CIA/vehicle animals between days 14 and 23 for the 30 mg/kg/day brensocatib group; between days 14 and 16 for the 3.0 mg/kg/day brensocatib group; and even the lowest dosage showed a significant reduction at day 18 (**Figure 2C**). Thus, brensocatib treatment improved all disease parameters tested at the two highest dosages in the rat CIA model.

**Figure 3:**
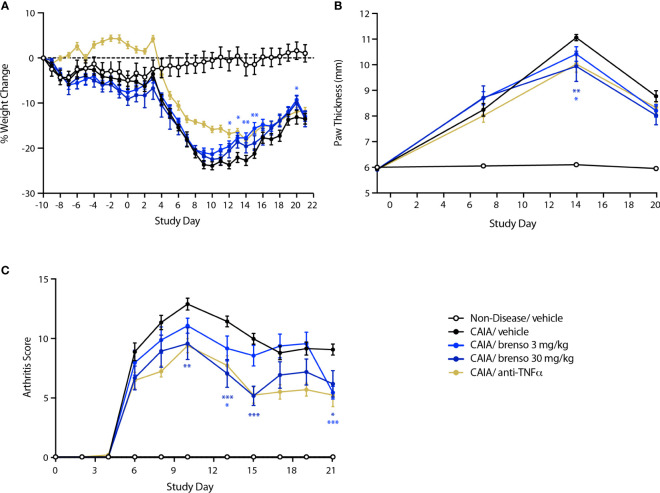
Effect of brensocatib on body weight and disease parameters in the mouse CAIA model. Mice were treated as described in **Figure 1** for arthritis induction and inhibitor administration. The following parameters were monitored: **(A)** body weight; **(B)** paw thickness; **(C)** clinical score (sum of the two hind paws). Mean ± SEM of 12 animals per group, except for non-diseased mice (5 animals). Statistical differences were determined using multiple t test comparisons and a false discovery rate of 1% as determined by the two-stage set-up method of Benjamini, Krieger, and Yekutieli. *p< 0.05; **p< 0.01; ***p< 0.001 relative to diseased animals receiving vehicle only.

We also explored the effect of brensocatib in another commonly used animal RA model – mouse CAIA. In this model, RA was induced by one collagen antibody immunization followed by LPS stimulation on day 3. Brensocatib treatment (at 3.0 mg/kg/day or 30 mg/kg/day) was initiated 10 days before collagen antibody immunization and lasted for 30 or 31 days. Disease levels were evaluated throughout the study. As shown in **Figure 3A**, treatment with brensocatib resulted in a partial reversal of body weight loss which reached statistical significance between days 12 and 15 for the 3.0 mg/kg/day dosage compared to diseased animals treated with vehicle. By comparison, the anti-TNFα group showed significant improvement in body weight throughout most of the study period. As shown in **Figure 3B**, treatment with brensocatib or anti-TNFα positive control had a modest though significant effect on inflamed paw volume only at day 14. By contrast, clinical scores were much improved in animals receiving the highest brensocatib dosage between days 10 and 15, and on day 21; similarly, the lower brensocatib dosage showed significant differences at days 13 and 21, relative to the vehicle/CAIA group (**Figure 3C**). Thus, brensocatib treatment improved most parameters tested in this model as well.

### Effect of brensocatib on histopathological parameters and neutrophil infiltration in mouse arthritic joints

**Figure 4:**
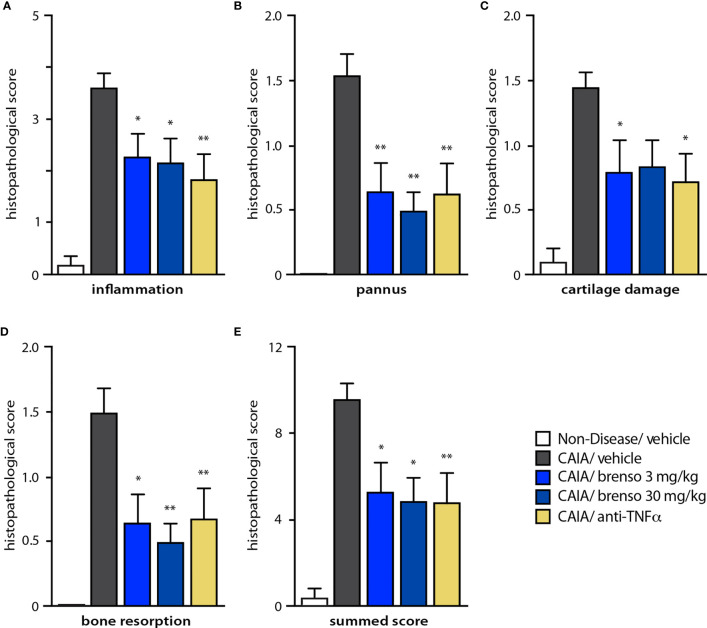
Effect of brensocatib on histopathology parameters in the mouse CAIA model. Mice were treated as described in **Figure 1** for arthritis induction and inhibitor administration. **(A-D)** Various histopathological scores were assessed on day 21; a summed score was also compiled **(E)**. Mean ± SEM of at least 10 animals per group, except for non-diseased mice (5 animals). Statistical differences were determined using Kruskal-Wallis test with Dunn’s multiple comparisons *post-hoc* test. *p< 0.05; **p< 0.01 relative to diseased animals receiving vehicle only.

**Figure 5:**
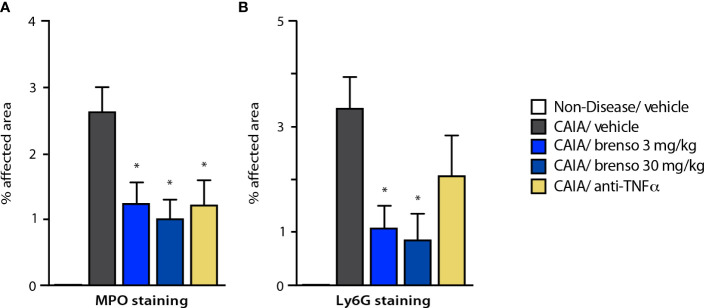
Effect of brensocatib on neutrophil infiltration in the mouse CAIA model. Mice were treated as described in **Figure 1** for arthritis induction and inhibitor administration and neutrophil infiltration was assessed by immunohistochemistry on day 21, using either **(A)** myeloperoxidase (MPO); or **(B)** Ly6G as readouts. Mean ± SEM of at least 10 animals per group, except for non-diseased mice (5 animals). Statistical differences were determined using Kruskal-Wallis test with Dunn’s multiple comparisons *post-hoc* test. *p< 0.05 relative to diseased animals receiving vehicle only.

To better understand how brensocatib treatment led to an improved disease score, we conducted histopathological analyses of arthritic joints; we also evaluated neutrophil infiltration into the joints since DPP-1 and its targets, the NSPs, are mainly or exclusively expressed in these immune cells. As shown in **Figures 4A–D** (dark gray bars) and **Figure S1**, all diseased mouse groups demonstrated histopathology consistent with the induction of arthritis, relative to naïve, healthy animals. Brensocatib treatment at both dosages significantly diminished almost all histopathological scores, with a potency similar to that of anti-TNFα antibodies (**Figures 4A–D**). A similar pattern was observed when we compiled the summed histopathology score (**Figure 4E**), for which treatment with brensocatib decreased the score by 51% at the 30 mg/kg/day dosage or by 41% at the 3.0 mg/kg/day dosage; this effect was comparable to treatment with an anti-TNFα antibody (49%). In the case of IHC analyses conducted using neutrophil markers, scores for MPO immunostaining were significantly decreased by 53% and 62% in mice given brensocatib at 3.0 or 30 mg/kg/day, respectively, or by 53% in animals receiving anti-TNFα antibody (**Figures 5A** and **S3**). Similarly, immunostaining for Ly6G was significantly decreased by 67% and 74% in mice given brensocatib at 3.0 or 30 mg/kg/day, respectively, versus only 38% in animals receiving the anti-TNFα antibody (**Figures 5B** and **S2**).

## Discussion

In this study, we assessed the efficacy of a potent DPP-1 inhibitor, brensocatib, in two well established RA models (rat CIA and mouse CAIA) which reproduce many of the pathophysiological features of the disease in humans (30). We previously evaluated various brensocatib doses in various rat and mouse strains and measured its effect on NSP activities in the bone marrow (31) as pharmacodynamic studies have established that this is where brensocatib initially acts (32) and provides better sensitivity than measuring NSP activities in blood. This led to the selection of brensocatib dosages of 3 and 30 mg/kg/day in the rodent models used herein. In both models, we confirmed that NSP activities were generally increased in diseased animals and brensocatib (at 3.0 and 30 mg/kg/day) restored NSP activity levels to baseline or below. In doing so, brensocatib was comparable to, or more effective than, dexamethasone in the rat CIA model; likewise, brensocatib proved generally more effective than anti-TNFα antibodies in the mouse CAIA model at both dosages. Thus, brensocatib inhibited DPP-1 substrates in these RA models, as was also observed in another neutrophil-mediated disorder, NCFBE, in humans (25).

Because elevated levels of active NSPs (whether secreted or NET-bound) contribute to the pathogenesis of several neutrophil-driven conditions, inhibiting upstream DPP-1 represents an opportunity for therapeutic intervention. We therefore investigated the effect of brensocatib on clinical endpoints (including body weight, paw volume or thickness, and arthritis/clinical score) in two animal models of RA. We found that brensocatib treatment improved all parameters tested at the highest dosage in the rat CIA model whereas significant improvements in some parameters were also observed using the intermediate dosage. In the mouse CAIA model, the DPP-1 inhibitor either improved disease parameters or they tended towards improvement; here again, the highest dosage (30 mg/kg/day) yielded the best improvements in arthritis score, rivaling the effect of an anti-TNFα antibody. We moreover found that treatment with the DPP-1 inhibitor markedly diminished both the histopathological score and neutrophil infiltration into arthritic joints in the CAIA mouse model, proving at least as good as the anti-TNFα antibody in doing so. In this regard, a direct effect of brensocatib towards neutrophil migration can most likely be ruled out, since neutrophils from DPP-1-deficient mice exhibit normal *in vitro* chemotaxis (23). Likewise, we found that in C57 mice, brensocatib did not affect neutrophil chemotaxis (D. Li, unpublished data). Instead, the beneficial effect of brensocatib treatment at the actual inflammation site probably reflects the neutralization of NSP activities and of their amplification of inflammatory reactions mediated by neutrophils. Such a general attenuation of the inflammatory tonus would in turn affect neutrophil activation, including their migration into arthritic joints. Thus, brensocatib may in effect break a part of the self-amplifying loop that is typical of early and ongoing inflammatory reactions.

Collectively, our data are consistent with the previous demonstration that DPP-1 knockout animals are resistant to the development of CIA and CAIA (24), and with a recent study that showed the anti-arthritic activity of a synthetic inhibitor of DPP-1, IcatC_XPZ-01_, in a CAIA mouse model (33). Our data also extend these findings by showing that DPP-1 inhibition reduces inflammation and neutrophil infiltration in mouse arthritic joints. This raises the possibility that brensocatib may represent a valuable therapeutic tool not only in RA, but in other chronic inflammatory disorders featuring a strong neutrophil component. In this regard, we found that brensocatib indeed attenuates lupus nephritis in a mouse model (34). More importantly, brensocatib inhibited NSP activity in both the blood and sputum of NCFBE patients in a six-month Phase 2 clinical trial (35); this reduction in sputum NSP activity was moreover associated to a prolonged time to first pulmonary exacerbation and an overall reduction in the frequency of such exacerbations (25, 35). Brensocatib is likewise being investigated in a Phase III clinical trial in NCFBE (NCT04594369). Future studies will also need to evaluate how brensocatib might be combined with existing treatments of RA to improve their efficacy given that even in patients responding to an ongoing treatment, improvement scores vary between 20-75% (www.arthritis.org). In this regard, the accumulated clinical experience in NCFBE and CF trials may provide supporting data for a future clinical translation of brensocatib in the treatment of RA.

## Data availability statement

The raw data supporting the conclusions of this article will be made available by the authors, without undue reservation.

## Ethics statement

The animal study was approved by Institutional Animal Care and Use Committee (IACUC) of Charles River Laboratories and the IACUC of Biomodels Inc., following the guidance of the Association for Assessment and Accreditation of Laboratory Animal Care (AAALAC). The study was conducted in accordance with the local legislation and institutional requirements.

## Author contributions

Conceptualization: JZ, JB and DC. Methodology: JZ, JB, DLa and DLi. Formal Analysis: PM, FL, JZ, JB, DLa, DLi and K-JC. Resources: WP. Data Curation: JB, DLa and DLi. Writing – Original Draft Preparation: PM and FL. Writing – Review & Editing: PM, DC, FL, JZ, JB, DLa, DLi, K-JC, and WP. Supervision: DC, WP. Project Administration: WP. All authors contributed to the article and approved the submitted version.

## Supplementary material

Supplementary Figure 1 Representative photomicrographs of histopathology analyses in the mouse CAIA model. Mice were treated as described in for arthritis induction and inhibitor administration. Tissues were taken at study end (d21) from the hind paws and processed for hematoxylin and eosin (H&E) analyses. **(A)** Paw from a non diseased animal treated with vehicle (with the approximate mean summed score for the group) has no detectable lesions. Higher magnification also shows no lesions. **(B)** Paw from an arthritic animal treated with vehicle only (with the approximate mean summed score for the group) has overall moderate inflammation and moderate cartilage damage with mild pannus, bone resorption, and periosteal bone forma tion. Arrow indicates a representative affected joint. Higher magnification of an affected joint shows representative synovitis, cartilage, and bone lesions. **(C)** Paw from an arthritic animal treated with 30 mg/kg brensocatib (with the approximate mean summed score for the group) has overall mild inflammation and cartilage damage with very minimal pannus and bone resorption in two digit joints. Arrows identify representative, minimally affected joints. Higher magnification of an affected joint shows very minimal synovitis and no cartilage or bone lesions. **(D)** Paw from an arthritic animal treated with 5 mg/kg of anti-TNFα (with the approximate mean summed score for the group) has very minimal lesions in the digit joints (not visible at 16x magnification). Arrow identifies a representative affected joint. Higher magnification of an affected joint shows very minimal synovial inflammation and no cartilage or bone changes.

Supplementary Figure 2 Representative photomicrographs of immunohistochemistry analyses in the mouse CAIA model. Mice were treated as described in for arthritis induction and inhibitor administration. Tissues were taken at study end (d21) from the hind paws and processed for Ly6G staining. **(A)** Paw from a non diseased animal treated with vehicle (with the approximate mean summed score for the group) has no Ly6G staining, even at higher magnification. **(B)** Paw from an arthritic animal treated with vehicle only (with the approximate mean summed score for the group) has mild Ly6G staining in affected joints. Higher magnification of an affected joint shows representative Ly6G staining in synovium and exudate. **(C)** Paw from an arthritic animal treated with 30 mg/kg brensocatib (with the approximate mean summed score for the group) has very minimal Ly6G staining (not visible at 16x magnification). Higher magnification of an affected joint shows representative Ly6G staining in synovium (arrow). **(D)** Paw from an arthritic animal treated with 5 mg/kg of anti-TNFα (with the approximate mean summed score for the group) has no Ly6G staining. Higher magnification of an affected joint shows no Ly6G staining.

Supplementary Figure 3 Representative photomicrographs of immunohistochemistry analyses in the mouse CAIA model. Mice were treated as described in for arthritis induction and inhibitor administration. Tissues were taken at study end (d21) from the hind paws and processed for myeloperoxidase (MPO) staining. **(A)** Paw from a non diseased animal treated with vehicle (with the approximate mean summed score for the group) has no MPO staining, even at higher magnification. **(B)** Paw from an arthritic animal treated with vehicle only (with the approximate mean summed score for the group) has marked MPO staining in affected joints. Higher magnification of an affected joint shows representative MPO staining in synovium and exudate. **(C)** Paw from an arthritic animal treated with 30 mg/kg brensocatib (with the approximate mean summed score for the group) has minimal MPO staining (not visible at 16x magnification). Higher magnification of an affected joint shows representative MPO staining in synovium (arrow). **(D)** Paw from an arthritic animal treated with 5 mg/kg of anti-TNFα (with the approximate mean summed score for the group) has very minimal MPO staining (not visible at 16x magnification). Higher magnification of an affected joint shows representative MPO staining in bone marrow.
