# Supplementary figures and images for: Brensocatib (an oral, reversible inhibitor of dipeptidyl peptidase-1) attenuates disease progression in two animal models of rheumatoid arthritis
Source: Front Immunol. 2023 Aug 11;14:1231047. doi: 10.3389/fimmu.2023.1231047 (PMC10451067; doi:10.3389/fimmu.2023.1231047)

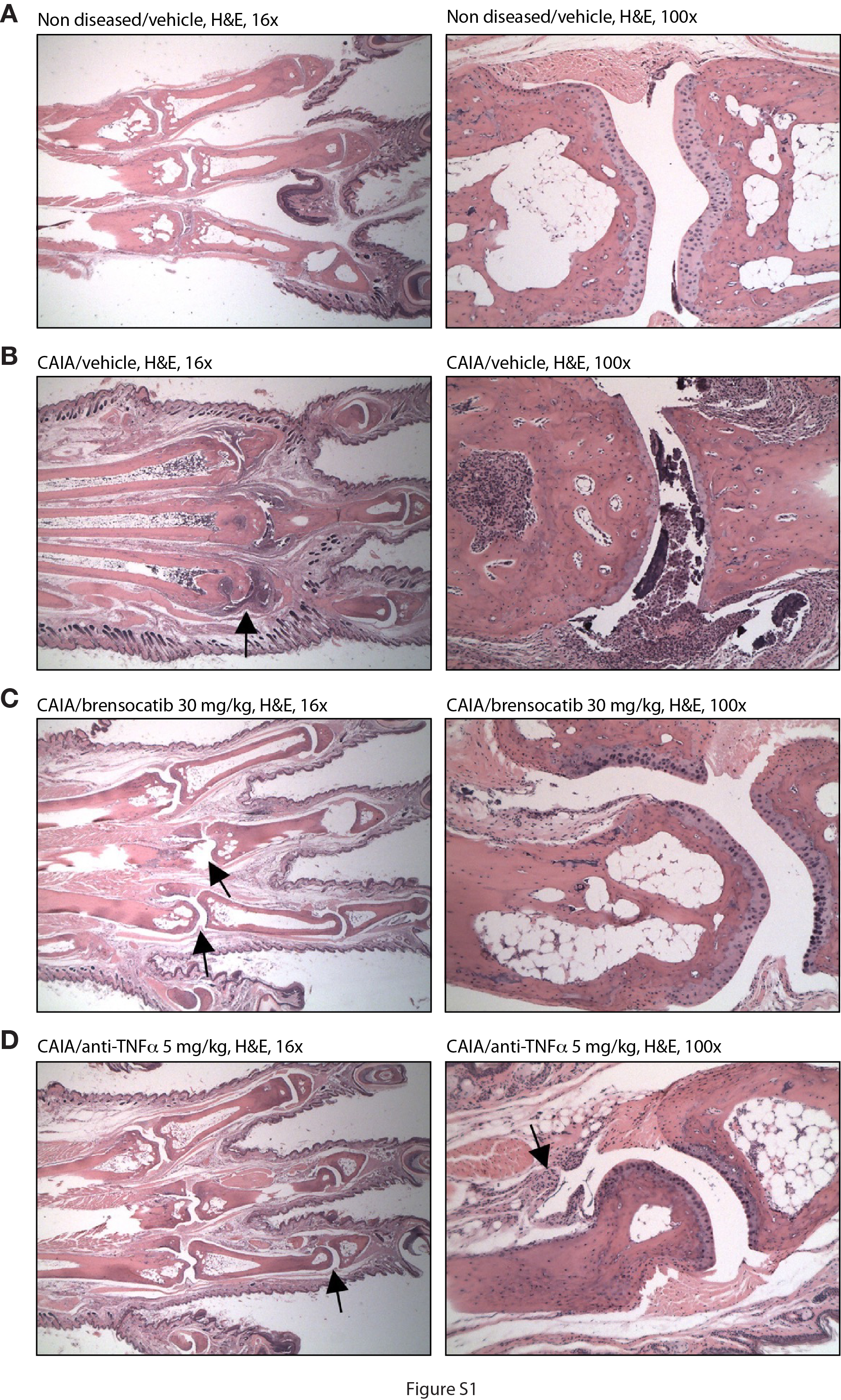

Supplement: Supplementary Figure 1 — Representative photomicrographs of histopathology analyses in the mouse CAIA model. Mice were treated as described in for arthritis induction and inhibitor administration. Tissues were taken at study end (d21) from the hind paws and processed for hematoxylin and eosin (H&E) analyses. (A) Paw from a non diseased animal treated with vehicle (with the approximate mean summed score for the group) has no detectable lesions. Higher magnification also shows no lesions. (B) Paw from an arthritic animal treated with vehicle only (with the approximate mean summed score for the group) has overall moderate inflammation and moderate cartilage damage with mild pannus, bone resorption, and periosteal bone forma tion. Arrow indicates a representative affected joint. Higher magnification of an affected joint shows representative synovitis, cartilage, and bone lesions. (C) Paw from an arthritic animal treated with 30 mg/kg brensocatib (with the approximate mean summed score for the group) has overall mild inflammation and cartilage damage with very minimal pannus and bone resorption in two digit joints. Arrows identify representative, minimally affected joints. Higher magnification of an affected joint shows very minimal synovitis and no cartilage or bone lesions. (D) Paw from an arthritic animal treated with 5 mg/kg of anti-TNFα (with the approximate mean summed score for the group) has very minimal lesions in the digit joints (not visible at 16x magnification). Arrow identifies a representative affected joint. Higher magnification of an affected joint shows very minimal synovial inflammation and no cartilage or bone changes. [file Image_1.jpeg]

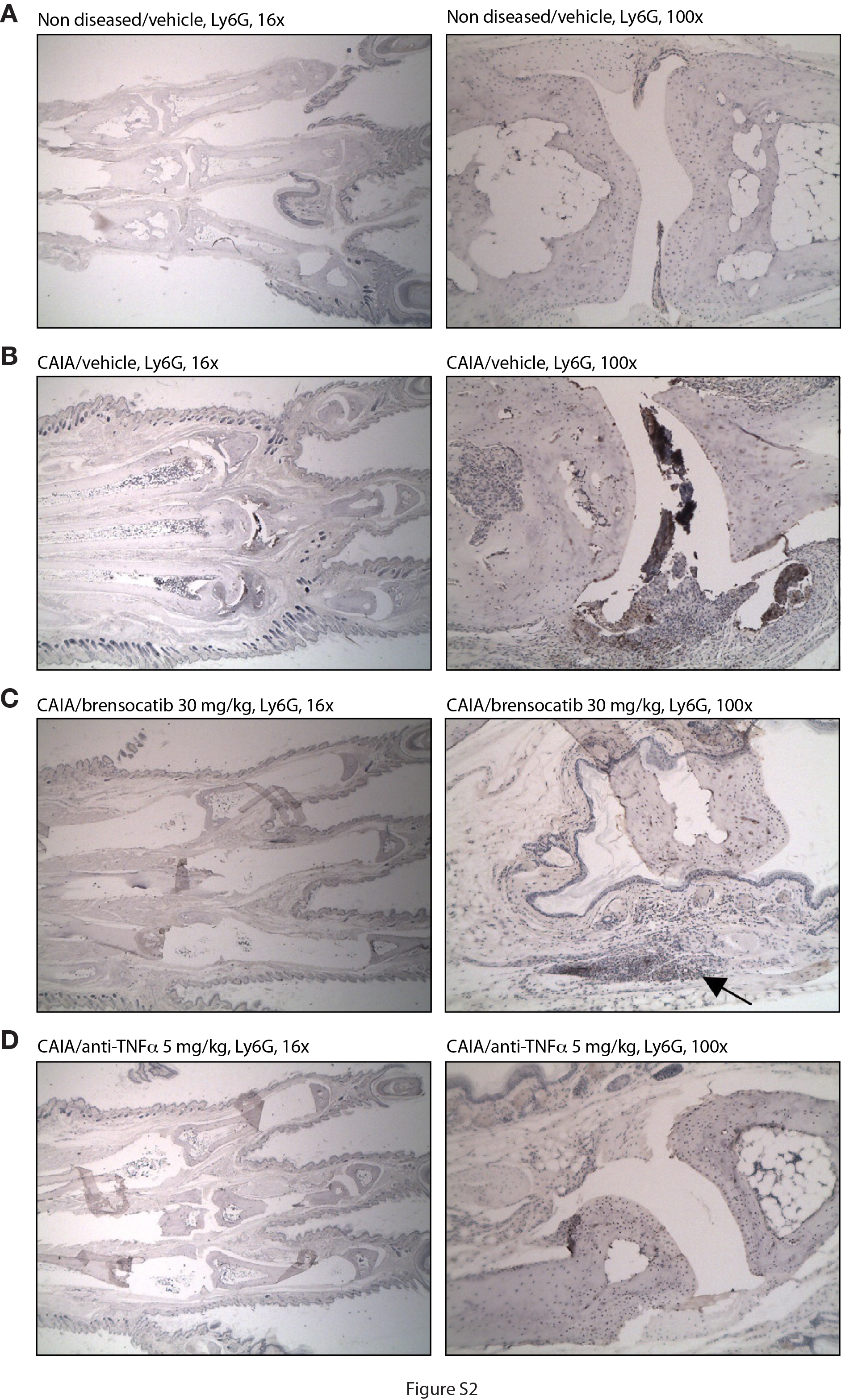

Supplement: Supplementary Figure 2 — Representative photomicrographs of immunohistochemistry analyses in the mouse CAIA model. Mice were treated as described in for arthritis induction and inhibitor administration. Tissues were taken at study end (d21) from the hind paws and processed for Ly6G staining. (A) Paw from a non diseased animal treated with vehicle (with the approximate mean summed score for the group) has no Ly6G staining, even at higher magnification. (B) Paw from an arthritic animal treated with vehicle only (with the approximate mean summed score for the group) has mild Ly6G staining in affected joints. Higher magnification of an affected joint shows representative Ly6G staining in synovium and exudate. (C) Paw from an arthritic animal treated with 30 mg/kg brensocatib (with the approximate mean summed score for the group) has very minimal Ly6G staining (not visible at 16x magnification). Higher magnification of an affected joint shows representative Ly6G staining in synovium (arrow). (D) Paw from an arthritic animal treated with 5 mg/kg of anti-TNFα (with the approximate mean summed score for the group) has no Ly6G staining. Higher magnification of an affected joint shows no Ly6G staining. [file Image_2.jpeg]

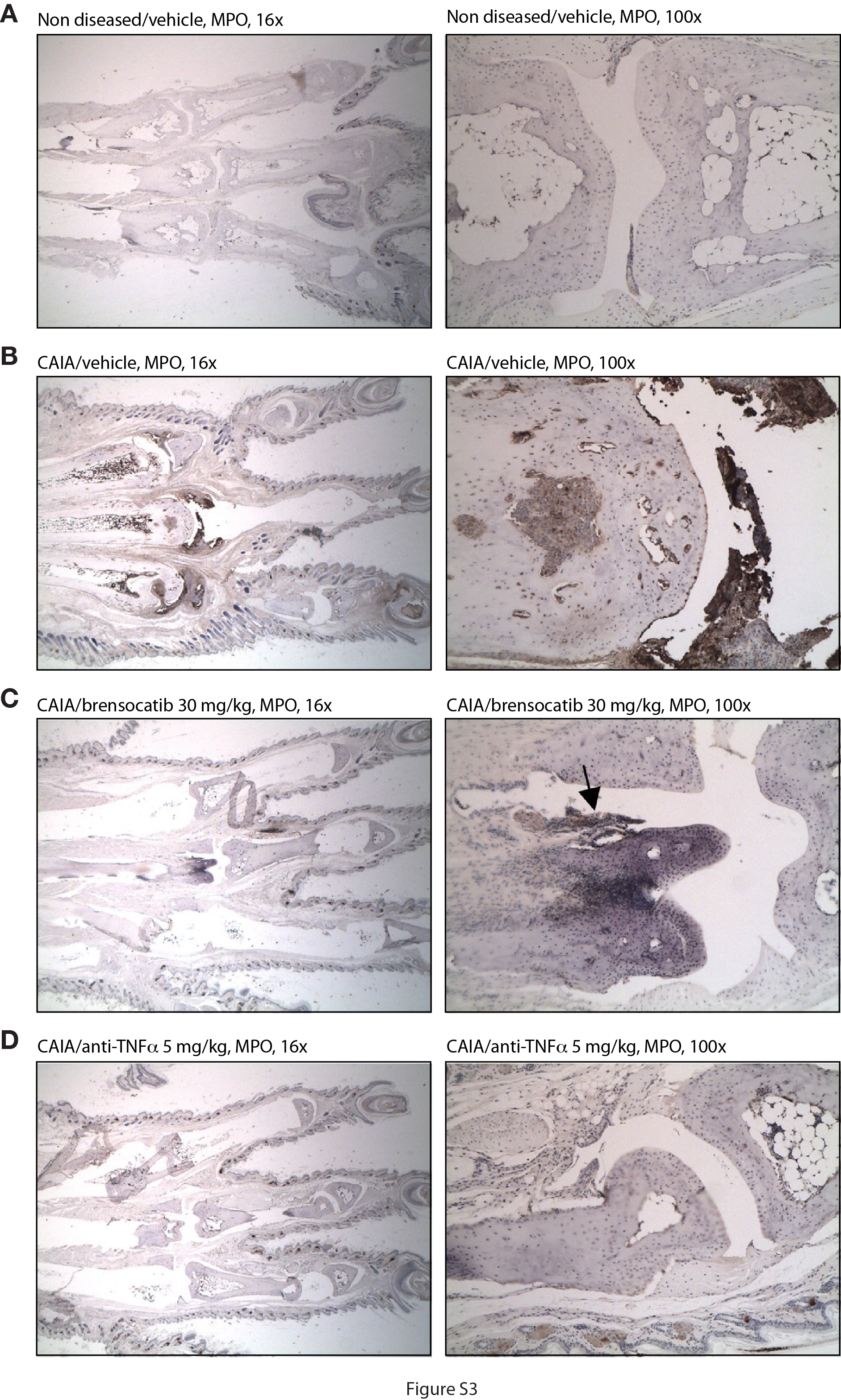

Supplement: Supplementary Figure 3 — Representative photomicrographs of immunohistochemistry analyses in the mouse CAIA model. Mice were treated as described in for arthritis induction and inhibitor administration. Tissues were taken at study end (d21) from the hind paws and processed for myeloperoxidase (MPO) staining. (A) Paw from a non diseased animal treated with vehicle (with the approximate mean summed score for the group) has no MPO staining, even at higher magnification. (B) Paw from an arthritic animal treated with vehicle only (with the approximate mean summed score for the group) has marked MPO staining in affected joints. Higher magnification of an affected joint shows representative MPO staining in synovium and exudate. (C) Paw from an arthritic animal treated with 30 mg/kg brensocatib (with the approximate mean summed score for the group) has minimal MPO staining (not visible at 16x magnification). Higher magnification of an affected joint shows representative MPO staining in synovium (arrow). (D) Paw from an arthritic animal treated with 5 mg/kg of anti-TNFα (with the approximate mean summed score for the group) has very minimal MPO staining (not visible at 16x magnification). Higher magnification of an affected joint shows representative MPO staining in bone marrow. [file Image_3.jpeg]
